# COVID-19 and Artificial Intelligence: the pandemic pacifier

**DOI:** 10.3126/nje.v10i4.33334

**Published:** 2020-12-31

**Authors:** Indrajit Banerjee, Jared Robinson, Abhishek Kashyap, Poornasha Mohabeer, Brijesh Sathian

**Affiliations:** 1-4 Sir Seewoosagur Ramgoolam Medical College, Belle Rive, Mauritius; 5 Geriatric and long term care Department, Rumailah Hospital, Hamad Medical Corporation, Doha, Qatar

## Background

COVID-19 remains a threat to the entire world [1, 2]. In an attempt to curb its spread and facilitate its treatment, the technological tool that is Artificial Intelligence (AI) is being researched as a potential alternative to conventional methods.

Profound social phenomena, i.e., globalism in combination with urban sprawl, population expansion and demographic changes, have profoundly altered the planet. Industrial Revolution 4.0 marks the dawn to the combination of digital, physical and biological systems, by application of digital skills such as Blockchain, Internet of things, Artificial Intelligence and Big data [3, 4]. This marks an empowering and inspiring era for medicine and science, where these technologies can automate interconnected ecosystems to enhance the experience of caregiving systems. This enabling us to analyze real-time data, model risk associations and predict future trends [5]. The application of this technology in numerous healthcare and medical fields acting as a decision assistance tool will provide aid from diagnosis to prognosis [6]. For example, detecting a myocardial infarction using a six lead ECG with a validated deep learning bases AI algorithm [7].

The application of these technologies is to minimize the loss caused by this pandemic. [8,9]. The outbreak of COVID19 was detected by the AI technology through a Toronto based startup, named Blue Dot. By using a surveillance programme supported by AI, they were able to unmask this outbreak prior to the Chinese Authorities and International Agencies. Another monumental example of modern-day technologies is the breakthrough in the treatment of Rheumatoid Arthritis by discovering a biologic named Baricitinib which is used in combination with Remdesivir [10].

For instance, AI tools in SARS-COV-2 pandemic are highly competitive to human performance, such as rapid screening and diagnosis of the disease, surveilling the efficacy of the treatment, keeping record and depicting active cases and mortality, inventions of medications and vaccines, relieving the workload of healthcare workers and extinguishing the spread of the disease. [11]. Contact tracing platforms like Aarogya Setu App [12], implemented by the Government of India, Australian Government's COVID Safe app [13], Trace Together- a Bluetooth-based contact tracing app developed in Singapore [14]; based on syndromic mapping/surveillance technology [15] Such a transition represents a futuristic breakthrough, as it imminently optimizes advanced, open-access platforms to unlock their capacity to assist researchers in designing a diagnostic test for pathogens. Deployment of international and national responses to any outbreak are far more rapid and efficient [16].

The unprecedented situation caused by COVID-19 has made the implementation of telemedicine-based services a pivotal manner to uphold the treatment of patients [17]. It is vital for the amalgamation of technology and knowledge to be better prepared for the next pandemic [18].

### Clinical applications in COVID-19 Artificial intelligence aided screening for COVID-19 vs conventional methods

The first suggested application of AI in this current pandemic is the early detection, screening and diagnosis of the disease [11]. Massachusetts Institute of Technology (MIT) designed an Open Voice Medicine model architecture, which makes use of forced-cough and other biomarkers, namely muscular degeneration and alterations in vocal cords to diagnose COVID19 [19]. Furthermore, it has been suggested to be a far better means of screening as compared to thermometers, which are not reliable in asymptomatic afebrile COVID19 positive patients. This proposed technology, with an accuracy of 98.5%, reaches 100% diagnosis of both symptomatic and asymptomatic COVID19 patients [19].

### Large scale application technologies

Digital services such as hotlines and online questionnaires have been extensively used [20]. These methods have their shortcomings in terms of hotlines being inundated. AI has allowed for the creation of symptom-to-disease search engines, one of which is named Symptoma [20]. The latter precisely identifies positive cases of COVID19 from a database of 20,000 other possible causes. Its accuracy is 96.32%. Moreover, its availability in 36 different languages lends itself to extensive use. Another symptom analyzer known as Isabel Healthcare, provides only 6000 differential diagnoses [20]. Similarly, the clinical use of AI guided chatbots has been established to ease out communication with the public [5, 21].

### RT-PCR and artificial intelligence combination

Owing to its increased availability, RT-PCR is the gold standard in diagnosis. CT scans have diagnosed patients in RT-PCR negative cases [22]. An AI based CT diagnosis, which demonstrates an accuracy level 95%, proves to have a promising role in the diagnosis of the disease. On a gross spectrum the accuracy was 90.8%, which is superior to RT-PCR with a sensitivity of 60-70% [22]. This study highlights the need for a combination of RT-PCR and CT scans in the detection of COVID19. [23].

### Rapidity of Artificial intelligence in diagnosis

As per Petrova V, a virologist from the University of Cambridge, United Kingdom, the value of AI in the diagnosis of COVID19 can be gauged from its ability to relieve the burden of clinicians during this pandemic. Applications such as Infervision’s AI can read the scan in 10 seconds as compared to a CT scan manual which takes up 15 mins of reading time [24].

Chest imaging (X-ray) can also be employed for the detection of COVID19 as performed in Beijing Hospital [5]. This approach has been put to practice in Zhongshan Ophthalmic Eye Center, China [5].

Contact tracing of individuals and minimizing the spread of COVID19 has been made possible by AI. In China, machine learning models were created from data, collected through mobile payment applications, migration maps and social media, the aim of which was to gather real-time information about the movement of people, who had been to Wuhan market. These models were then used to predict the transmission dynamics of COVID-19 and hence curb its spread [25]. Lastly AI has contributed extensively in new drug design and drug repurposing for COVID19 [26, 27].

### Current status of Artificial intelligence

The principle and core basis of artificial intelligence is the analyses of historical data [28]. A pitfall of this heavy reliance on historical data suggests that a wide spectrum grassroot implementation across all sectors will face many challenges as in many cases new system development cycles will need to be procured and initiated, thus retarding the process. In juxtaposition to this the pre-existing intrinsic technological and digital base lends itself to be retrofitted to support further artificial intelligent modifications to curb the spread of the virus via the formation of an integrated digital ecosystem [29, 30].

Future scope of Artificial intelligence:

The future endorsement and wide scope application of artificial intelligence has never been so clear cut and supported [31]. The development of software and technology to improve screening, mapping, data collection and future predictions of outbreaks is well underway and various models are currently being implemented and tested in across the globe [32]. It is evident that raw data collection from pre-existing social media applications and or newly designed platforms will be key for the real-time assessment of the situation. The use of one’s mobile cellular device as a continuous data feed and point of input will thereby allow projections of outbreaks and epicenters to be more accurate and thus will form the basis of future models. The future use of this artificial intelligent data driven model is the donnée to all current research being undertaken and will form the foundation of the worlds future preparations to combat pandemics to the likes of COVID19 in future [5].

## Conclusion

Artificial intelligence will become a mainstay in both the diagnosis and treatment of COVID-19 as well as similar pandemics in future. The application and system development will be challenging; the accuracy and rapidity of its use far outweigh this drawback. The current global technological leaders have proven that the retro modification of current data systems and applications have been indispensable in the war on COVID-19, thus permanently securing their development and application in future.
